# An epidemiological study of prevalence and comorbidity of non-clinical Dyslexia, Dysgraphia and Dyscalculia symptoms in Public and Private Schools of Pakistan

**DOI:** 10.12669/pjms.36.7.2486

**Published:** 2020

**Authors:** Farzana Ashraf, Najma Najam

**Affiliations:** 1 Farzana Ashraf, COMSATS University Islamabad, Lahore Campus, Pakistan; 2 Najma Najma, Institute of Applied Psychology, University of The Punjab, Lahore, Pakistan

**Keywords:** Dyslexia, Dysgraphia, Dyscalculia, Gender, Specific learning disabilities (SLD) symptoms

## Abstract

**Objective:**

Dealing with Dyslexia, Dysgraphia and Dyscalculia symptoms is a major challenge for teachers and school psychologists while addressing students’ issues. The present study was designed to examine the prevalence and comorbidity of specific learning disabilities (SLD) symptoms such as dyslexia, dysgraphia and dyscalculia in public and private schools of Lahore, Pakistan.

**Methods:**

This cross-sectional study was conducted in four schools of Lahore from June, 2019 to December 2019. We examined 666 participants (boys= 384, girls= 282) from two public (n=409) and two private (n=257) schools of Lahore with a mean age of 13 years (SD±1.44). Participants were assessed on Learning Disabilities Checklist (LDC) along with a demographic sheet. The data were analyzed by using descriptive statistics (frequencies and percentages) and inferential analyses of Chi Square test of association and Cohen’s Kappa by using SPSS version 24.

**Results:**

Findings indicated that 39% participants showed SLD symptoms, 33% dyslexia, 48% dysgraphia and 45% dyscalculia symptoms. Significant co-morbidities were seen, such as 30% for dyslexia and dysgraphia symptoms, dyslexia and dyscalculia 26% and dysgraphia and dyscalculia as 36%. Variations in SLD, dyslexia, dysgraphia and dyscalculia symptoms were also seen across gender and schools with significant higher prevalence in public schools.

**Conclusion:**

High prevalence of SLD symptoms and comorbidity in students was found which is alarming, particularly in public sector schools in Pakistan. SLD and dyslexia were higher for boys, whereas girls scored high on dysgraphia and dyscalculia. Therefore, there is great need of introducing screening measure of assessment of SLD and management strategies to deal with these issues.

## INTRODUCTION

Specific Learning Disabilities (SLD) are a heterogeneous group of neuro-behavioral features demonstrated by significant persistent, specific and unexpected difficulties in the acquisition and use of efficient reading (dyslexia), writing (dysgraphia) and mathematical (dyscalculia) abilities. These difficulties are experienced independent of adequate social, cultural opportunities, motivation level, intelligence, intact senses and conventional instructions.^1^ Broadly speaking SLD are widely examined in clinical and sub-clinical groups (e.g., With ADHD, Autism and SLD) but insufficiently tested in normative samples. While in the local context, no empirical evidence or latest updates are available yet previous verbal and non-empirical reports estimate a 10 to 18 % prevalence of SLD symptoms in Pakistani school children; irrespective of academic level.^2^ Due to variant features linked with each difficulty, the prevalence of these symptoms also varies. Among these, dyslexia is most commonly occurring learning disorder affecting approximately 80% of the school children identified with SLD.^3^ In case of dysgraphia, available literature lacks to identify any specific direction, yet closely related writing deficits are estimated in a wide range of 5 to 20%.^4^ Followed by there is consistent agreement that about 5 to 8% of primary school children demonstrate symptoms of dyscalculia worldwide.^5^ For adolescent population, it remains unknown. In a south Asian normative sample (from East India), estimated prevalence for SLD, dyslexia, dysgraphia and dyscalculia were found as 15.17%, 11.2%, 12. 5%, and 10.5 % respectively.^6^

Past studies have widely reported independent prevalence of dyslexia, dysgraphia and dyscalculia arguing over variant features of these disabilities in diverse contexts, yet it remains interesting to explore co-morbidities in these demonstrations at different levels as all three difficulties are operated by common cognitive structures. A past study reported co-occurrence of dyslexia and dyscalculia as 10% irrespective of adequate learning environment and average intellifence.^7^ In context of comorbidity between dyslexia and dysgraphia, statistics represent more intense picture as 30 to 47% with reading difficulties also demonstrates writing problems.^8^

No empirical evidence is available reporting co-existence of dysgraphia and dyscalculia, yet a latest study documented 46% of co-occurrence of dyslexia and/or dysgraphia with dyscalculia.^9^ This also directs toward the notion that due to common cognitive and neurological functions, dysgraphia and dyscalculia may have possible comorbidity. In addition, how these features prevail differently across personal characteristics of students such as age, gender and school may also provide unique directions. Along with these personal/demographic characteristics in relation to prevalence of dyslexia, dysgraphia and dyscalculia, schools’ settings (e.g., public and private) in which students are encountering these disabilities is also important to be examined. In the context of comorbidity in local perspective, SLD is tested in relation to other psychiatric features such as depressive^10^ and anxiety symptoms yet co-occurrence of SLD are needed to be examined.

In Pakistani context, this area of research is comparatively less explored which could be due to poor understanding of chronicity of under investigation phenomenon and lack of students’ counseling and formal assessment services for screening of these disabilities. This paucity of research enforces need of structured and well-equipped psychological services to cater need of identification and management of these symptoms. Though it requires organized and structured efforts at larger scale, yet the present research is an effort to partially fulfill this gap. The present study examined the prevalence of the SLD and comorbidity of dyslexia, dysgraphia and dyscalculia.

## METHODS

In this cross-sectional study, conducted from June, 2019 to December 2019, 666 students (boys=384, girls=282) from two public (n=409) and two privates (n=257) schools of Lahore ages between 11 to 17 years (M=13.30 +1.44) were selected through random sampling technique. The sample size was determined by Qualtrics method of sample size calculated using with 5% of margin of error and 95% confidence interval. Learning Disabilities Checklist (LDC)^11^ was used in addition to the demographic questionnaire consisted of age, gender, and education level of participants. LDC comprised of 35 items measuring dyslexia (*15 items*), dysgraphia (*10 items*) and dyscalculia (*10 items*) on dichotomous scales (*yes=1. no=0*) with a score ranging from 0 to 35. LDC scores could be used as composite as well as in categories; high scores as indicators of more learning problems. LDC categorizes obtained scores below/at 25% as mild, between 26% to 49% as moderate and at/or 50% as severe. In the present study, alpha coefficients for SLD, dyslexia, dysgraphia and dyscalculia were calculated as 0.94, 0.87, 0.84 and 0.86 respectively. After ethical approval of research, formal permission was obtained from school administration and parents. Ethical approval was obtained from Ethical Review Board of Department of Humanities, COMSATS University, Lahore (CUI/LHR/HUM:632; dated July 1^st^, 2019). The ethical considerations include brief description of the subject study, confidentiality and privacy of provided information, withdrawing from research at any point during the study, and use of provided data only for research purpose. They were also informed about the objectives of research and implications of the study findings. To avoid the influence of any confounding factors, participants with any other significant neurological deficits or disabilities, significant behavioral issues or taking any counseling session were also excluded. School administration facilitated in screening of such participants. After collecting data, it was processed for further analysis through SPSS version 24.

## RESULTS

From sample of 666 students, 384 (58%) were boys and 282 (42%) were girls with a mean age of 13 years (*±*1.44). Of 666 participants, 409 (67%) were enrolled in public and 257 (37%) in private schools; this ratio is well proportionate to the overall ratio of boys to girls, number of public and private schools in Lahore and students enrolled in public and private schools. These participants were enrolled in grades 6^th^ to 10^th^; 21%, 23%, 16%, 24%, 16% respectively. Further analysis demonstrated the prevalence of mild, moderate and severe SLD prevailing as 36%, 25% and 39% respectively. For dyslexia, dysgraphia and dyscalculia, prevalence at severe level was estimated as 33%, 48% and 45% respectively. Along with total sample, the prevalence of SLD, dyslexia, dysgraphia and dyscalculia for boys and girls, public and private schools were also calculated. Table-I reveals that boys experienced comparatively more severe SLD (boys=44% and girls=41%) and dyslexia (boys=34% and girls=32%) than girls. Whereas dysgraphia (49% and 47% respectively) and dyscalculia (46% and 44% respectively) were comparatively high for girls than boys.

**Table-I:** Frequencies and Percentages of SLD across Gender, School and Age Groups.

Measures		Total sample (n=666)	Boys (n=384)	Girls (n=282)	Public Schools (n=409)	Private Schools (n=257)
Specific Learning Disabilities	Mild	242(36%)	131(34%)	116(42%)	142(34%)	141(55%)
	Moderate	165(25%)	83(22%)	48(17%)	72(18%)	66(26%)
	Severe	259(39%)	170(44%)	114(41%)	195(48%)	50(19%)
Dyslexia	Mild	264(40%)	141(37%)	119(43%)	130(32%)	134(52%)
	Moderate	178(27%)	110(29%)	68(25%)	100(24%)	78(30%)
	Severe	224(33%)	133(34%)	91(32%)	179(44%)	45(18%)
Dysgraphia	Mild	231(34%)	122(32%)	105(38%)	108(26%)	123(48%)
	Moderate	118(18%)	81(21%)	37(13%)	69(17%)	49(19%)
	Severe	317(48%)	181(47%)	136(49%)	232(57%)	85(33%)
Dyscalculia	Mild	256(38%)	131(34%)	121(44%)	123(30%)	133(52%)
	Moderate	113(17%)	83(22%)	30(11%)	75(18%)	38(15%)
	Severe	297(45%)	170(44%)	127(46%)	211(52%)	86(33%)

While comparing participants in public and private schools, findings were clearer as students in public school out number students in private schools on SLD, dyslexia, dysgraphia and dyscalculia (Table-I). The cross prevalence of dyslexia, dysgraphia and dyscalculia are shown in Table-II. About 30% of participants with dyslexia also have symptoms of dysgraphia, while in case of dyscalculia, it was observed is 26%. In addition, 36% of students with dysgraphia symptoms also have dyscalculia symptoms. Chi square test of association demonstrated strong associations between all study constructs; Cohen’s kappa was also calculated to estimate the strength and agreement between categories; which supplemented the findings of Chi square test of association. Whereas, correlations were also obtained to support the findings from Chi square and Cohen’s Kappa. Cohen’s Kappa and correlation illustrated moderate effect size and relationships respectively.

**Table-II:** Cross prevalence of Dyslexia, Dysgraphia and Dyscalculia.

Measures		Dyslexia			χ	κ	r

		Mild	Moderate	Severe			
Dysgraphia	Mild	200(30%)	43(6%)	21(3%)	411.51^***^	0.52	0.73
	Moderate	25(4%)	55(8%)	98(15%)			
	Severe	6(1%)	20(3%)	198(30%)			
Dyscalculia	Mild	206(31%)	31(5%)	27(4%)	339.54^***^	0.48	0.66
	Moderate	32(5%)	54(8%)	92(14%)			
	Severe	18(3%)	28(4%)	178(26%)	
			*Dysgraphia*					
Dyscalculia	Mild	185(28%)	24(4%)	22(3%)	340.696^***^	0.51	0.68
	Moderate	44(7%)	39(6%)	35(5%)			
	Severe	27(4%)	50(8%)	240(36%)			

*** p<0.0001

## DISCUSSION

The current study estimated prevalence of SLD as 39% which is comparatively higher than in already estimated prevalence of 2% to 15 in south Asian study (e.g., India, 2011) and 2% to 17% in global general prevalence.^12^ This discrepancy could be due to the poor perception of distinction between SLD and low intellectual capacity in students. It could also be justified from a previous Pakistani study claiming dyslexia in relation with poor cognitive functioning.^13^ Though, some of the latest studies have highlighted the need of computerized assistance techniques facilitating students with dyslexia^14^, yet at large no structural system of students’ assessment has been formally introduced and implemented in public as well as private schools of Pakistan. As such early symptoms of SLD remain unidentified and therefore undiagnosed at school level as compare to the schools in India and Western countries.^15^

This study also found a significant cross prevalence of dyslexia, dysgraphia and dyscalculia which is very unique finding in the local context. Perhaps this is first study of inter prevalence of dyslexia, dysgraphia and dyscalculia. Earlier, cross prevalence of these symptomologies was assessed collectively as specific learning disabilities with co-occurrence of depressive symptoms^11^ and anxiety symptoms (only in girls’ sample).^16^ The present study findings are similar to the previous western studies^8^,^9^ demonstrating a comparative higher relevance of dyslexia than dysgraphia and dyscalculia.^3^ A possible explanation of this distinction could be variations in distribution of reading, writing and mathematical problem-solving tasks students are assigned formally; 80% of students’ academic activities engage reading skills.^10^ Some aspects of the study findings are a unique contribution; for example, significant cross prevalence of dysgraphia and dyscalculia as no previous study as per the documented literature has estimated this prevalence in normative samples. In addition to general prevalence, we also explored the prevalence across gender and schools. In current study findings indicated slight variations in prevalence of the SLD, dyslexia, dysgraphia and dyscalculia; e.g., SLD higher for boys and dysgraphia and dyscalculia for girls. Which is supplementing past mixed findings where the boys showed more dysgraphia symptoms than girls, while girls were more impaired in dyscalculia. No differences were seen in dyslexia and overall SLD.^17^ High rates of prevalence of dyslexia, dysgraphia and dyscalculia may be attributed to the lack of early assessment and screening strategies at school level which is also partially supported by a local finding claiming that there is no systematic research reflecting upon the presence of dyscalculia in Pakistani students.^18^ The findings also direct that SLD (dyslexia, dysgraphia, dyscalculia) is at the more crucial to identify than any other psychological hindrance particularly in school setting. A review of literature on medical students in the local context supported this notion by describing that SLD are found challenging not only for students but also teachers. Lack of awareness about its symptomology and manifestation makes it hard for teachers to report such cases to school psychologist and clinicians. As a result, it remains unidentified, therefore undiagnosed and consequently unmanaged.^19^ In continuation, a latest qualitative study focusing on remedial teaching approaches for students with dyslexia found that pre and follow-up assessments are intensively needed. Along with traditional approaches of teaching students with dyslexia, innovative approaches such as play therapy integrative approaches may be effective. Furthermore, students with dyslexia face stigmatization, slow progress, behavioral problems.^20^

### Limitations and Suggestions

Though the current study reported high ratio in prevalence of specific learning disabilities (SLD) than previously reported findings which could be attributed to the differences in assessment measures used and classification criterion for SLD between western literature and Pakistani context. Other reasons could be lack of proper awareness, insufficient comprehension of the concept of SLD in mainstream schools and paucity of counseling services or school psychologists. Further, high ratio could be balanced out by splitting severe into severe and profound categories. In addition, LDC is screening source of assessment, diagnostic tools may also be used to obtain a more refined picture. Furthermore, clinical control trial studies will not only add to the clinical knowledge but also will guide to design appropriate management strategies according to the academic level and background of students.

## CONCLUSION

The present study finding is a valuable addition to existing literature globally and in Pakistani context specifically. Further, the findings from this study are useful from clinical as well as academic aspects; guiding clinicians and school psychologists to outline clinical assessments and protocols at screening, diagnostic and management levels. In addition, on the basis of our findings, early identification and screening will also help clinicians to design more efficient and economical tools of assessment of dyslexia, dysgraphia and dyscalculia at individual as well as collective levels. The present study findings have not only revealed significant general prevalence of SLD as well as dyslexia, dysgraphia and dyscalculia but also examined the prevalence across gender and schools and cross comorbidity of dyslexia, dysgraphia and dyscalculia. In indigenous perspective, these findings set a benchmark for future researchers to explore these dimensions from more diverse perspectives. Further, there is a dire need to screen out SLD at very initial levels of schooling so that suitable management for SLD could be provided timely. Besides, a collective support of family, peers, teachers and clinicians may facilitate and strengthen process of identification, screening, diagnosis and management of SLD.
